# Quantitative ultrasonic assessment for detecting microscopic cartilage damage in osteoarthritis

**DOI:** 10.1186/ar1463

**Published:** 2004-11-16

**Authors:** Koji Hattori, Ken Ikeuchi, Yusuke Morita, Yoshinori Takakura

**Affiliations:** 1 Department of Orthopaedic Surgery, Nara Medical University, Kashihara, Nara, Japan; 2 Institute for Frontier Medical Sciences, Kyoto University, Kyoto, Japan

**Keywords:** cartilage, evaluation, osteoarthritis, ultrasound, wavelet transformation

## Abstract

Osteoarthritis (OA) is one of the most prevalent chronic conditions. The histological cartilage changes in OA include surface erosion and irregularities, deep fissures, and alterations in the staining of the matrix. The reversibility of these chondral alterations is still under debate. It is expected that clinical and basic science studies will provide the clinician with new scientific information about the natural history and optimal treatment of OA at an early stage. However, a reliable method for detecting microscopic changes in early OA has not yet been established. We have developed a novel system for evaluating articular cartilage, in which the acoustic properties of the articular cartilage are measured by introducing an ultrasonic probe into the knee joint under arthroscopy. The purpose of this study was to assess microscopic cartilage damage in OA by using this cartilage evaluation system on collagenase-treated articular cartilage *in vivo *and *in vitro*. Ultrasonic echoes from articular cartilage were converted into a wavelet map by wavelet transformation. On the wavelet map, the maximum magnitude and echo duration were selected as quantitative indices. Using these indices, the articular cartilage was examined to elucidate the relationships of the ultrasonic analysis with biochemical, biomechanical and histological analyses. In the *in vitro *study, the maximum magnitude decreased as the duration of collagenase digestion increased. Correlations were observed between the maximum magnitude and the proteoglycan content from biochemical findings, and the maximum magnitude and the aggregate modulus from biomechanical findings. From the histological findings, matrix staining of the surface layer to a depth of 500 μm was closely related to the maximum magnitude. In the *in vivo *study, the maximum magnitude decreased with increasing duration of the collagenase injection. There was a significant correlation between the maximum magnitude and the aggregate modulus. The evaluation system therefore successfully detected microscopic changes in degenerated cartilage with the use of collagen-induced OA.

## Introduction

Osteoarthritis (OA), also referred to as degenerative joint disease, is one of the most prevalent chronic conditions. It consists of a general progressive loss of articular cartilage, remodeling and sclerosis of the subchondral bone, and the formation of subchondral bone cysts and marginal osteophytes. In particular, the degenerative processes of articular cartilage can be accelerated by a single traumatic event, multiple repetitive loads, or local chemical and mechanical factors [1]. The histological changes that occur in cartilage in OA are a striking feature of the disease. The earliest alterations include surface erosion and irregularities, deep fissures and alterations in the staining of the matrix. The reversibility of these chondral alterations is still under debate [2]. It is expected that clinical and basic science studies will provide the clinician with new scientific information about the natural history and optimal treatment of OA at an early stage. However, a reliable method for detecting microscopic changes in early OA has not yet been established.

We previously developed a novel system for evaluating articular cartilage, in which the acoustic properties of articular cartilage are measured by introducing an ultrasonic probe into the knee joint under arthroscopy [3,4]. The analysis system is based on wavelet transformation of the reflex echogram from articular cartilage. In detail, reflex echograms from many articular cartilage samples were transformed into wavelet maps by wavelet transformation and examined in detail. The results revealed two quantitative parameters on the wavelet maps that could be used as indices for the quantitative assessment of articular cartilage, namely the maximum magnitude and the echo duration at the 95% interval of the maximum magnitude. Macroscopic articular cartilage degeneration would result in a decreased magnitude and prolonged echo duration, as indicated by the L-shaped distribution obtained with human cadaver cartilage. However, the point at which this system can detect microscopic changes in articular cartilage degeneration is unknown. If our evaluation system can detect microscopic changes in cartilage *in vivo *and *in vitro*, it may provide a means to solve the problem of whether or not microscopic damage in OA is reversible. Moreover, this system will provide new information about the natural history and treatment of OA.

The purpose of this study was to investigate the clinical usefulness of our system for evaluating microscopic damage in OA. We therefore evaluated articular cartilage with no visible disruption in collagenase-induced experimental OA, using our system to assess the microscopic damage. The present study was also performed to investigate the correlation between ultrasonic examination and biomechanical or biochemical examination. The goal of our study was to further elucidate the processes of articular cartilage degeneration with the use of our ultrasonic evaluation system.

## Materials and methods

### *In vitro *study

Pig osteochondral plugs (diameter 5 mm; *n *= 77) were prepared for this study. The pig cartilage was delivered intact within 6 hours of slaughter, and the knee joints were stored at less than -30°C until use. During the preparation, the knee joints were first thawed in saline at 20°C and the joint cartilage was then exposed. Osteochondral plugs were excised from a flat area of the cartilage with a metal punch. The osteochondral samples were subsequently digested in PBS (Invitrogen Corporation, Carlsbad, CA, USA) containing 30 U/ml collagenase type ΙΙ (Worthington Biochemical Corporation, Lakewood, NJ, USA) at 37°C for 1, 2, 4, 8, 16 and 24 hours. Cartilage samples in PBS alone at 37°C were used as controls. After digestion, all the samples in each group (*n *= 11) were examined by ultrasonic evaluation. Four samples in each group were prepared for mechanical testing by cartilage indentation. Four samples of each group were used for biochemical examination and were separated from the bone with the use of an autopsy saw. Three samples in each group were prepared for histological analysis.

### Ultrasonic analysis

**Figure 1:**
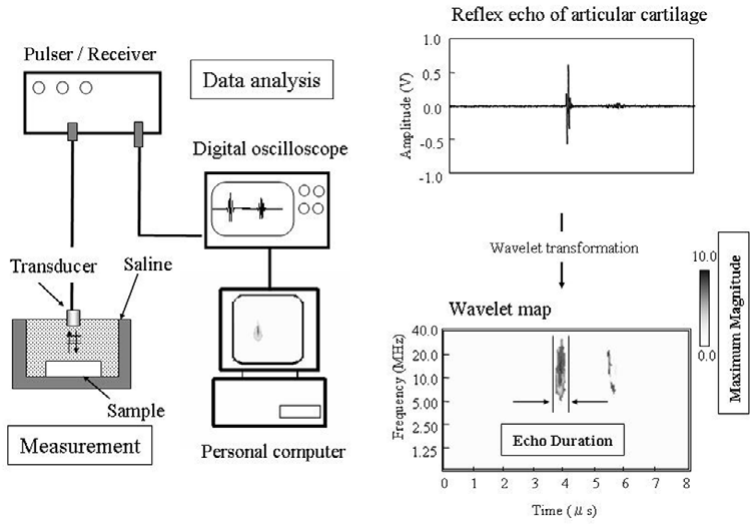
Schematic illustration of the articular cartilage analysis and measurement methods of the cartilage samples. A reflex echogram of articular cartilage and a wavelet map are shown. The maximum magnitude is indicated by the gray scale and the echo duration is defined as the length of time for which 95% of the echo signal is detected.

Our evaluation method was described in detail in a previous manuscript [3], and is illustrated in Fig. 1. In brief, during arthroscopic examination, ultrasonic evaluation was performed by using an ultrasonic probe with a transducer fixed to the tip. The transducer (Panametrics Japan Co. Ltd., Tokyo, Japan) was small (diameter 3 mm; thickness 3 mm) and used a flat ultrasonic wave (center frequency 10 MHz). Ultrasonic echoes from the cartilage surface were converted into a wavelet map by wavelet transformation. The wavelet transformation (*W*(*a*,*b*)) of the reflex echogram (*f*(*t*)) is expressed by



where Ψ(*t*) is the mother wavelet function.

For the mother wavelet function, Gabor's function was selected. The right side of Fig. 1 shows a typical ultrasonic echogram (upper) and wavelet map (lower) of an intact articular cartilage surface *in vitro*. The wavelet map shows a two-dimensional map whose *x*-axis and *y*-axis represent time and frequency, respectively, and the magnitude is indicated by the gray scale. As quantitative indices we used the maximum magnitude and echo duration, which was defined as the length of time that included 95% of the echo signal. These indices were calculated automatically by a computer. Articular cartilage was evaluated *in vivo *and *in vitro *with these two indices.

### Biochemical analysis

The cartilage samples were freeze-dried overnight after measuring the wet weight. The dry weight of the samples was then measured, and the amount of water was calculated. The water content of the cartilage was determined as a percentage by using the following equation: 100 × (wet weight - dry weight)/wet weight. The samples were digested with papain (Sigma Chemical Co., St Louis, MO, USA) (40 μg/ml in 20 mM ammonium acetate, 1 mM EDTA, 2 mM dithiothreitol) for 48 hours at 65°C and then stored at -20°C until analysis. Aliquots of the digests were assayed separately for the proteoglycan and collagen contents. The proteoglycan content was estimated by quantifying the amount of sulfated glycosaminoglycans with the use of a dimethylmethylene blue dye binding assay (Polyscience Inc., Washington, PA, USA) and spectrophotometry (wavelength 525 nm). A standard curve for the analysis was generated with bovine trachea chondroitin sulfate A (Sigma). The collagen content was estimated by determination of the hydroxyproline content. Aliquots of the papain digest were hydrolyzed at 110°C in 6 M HCl for 18 hours. The hydroxyproline content of the resulting hydrolyzate was determined by the chloramine-T/Ehrlich reagent assay and spectrophotometry (wavelength 561 nm). A standard curve for this analysis was generated with L-hydroxyproline (Sigma).

### Biomechanical analysis

A custom-made indentation testing device was used for mechanical testing to determine the creep and recovery behavior of the osteochondral samples. The samples were mounted on stainless steel plates with cyanoacrylate cement such that the rigid porous indenter tip was perpendicular to the test site on the cartilage surface. The porous indenter was made of titanium alloy particles (Ti-6Al-4V; diameter 75–180 μm). The porous permeable indenter tip (diameter 1.5 mm) was ultrasonically cleaned before testing to ensure ease of fluid flow from the specimen into the tip. The displacement of the indenter was measured using a laser measurement sensor (LB040/LB-1000; Keyence Corporation, Osaka, Japan). After equilibration under a tare load (0.0098 N), the test load (0.0098 N) was applied and the osteochondral specimen was allowed to creep to equilibrium. Equilibrium was determined as being when no further variations occurred in the observed creep value for 20 min. After creep equilibrium had been achieved, the test site was unloaded and the recovery was observed. The cartilage thickness was then measured at an exact location and orientation site with a penetrating steel needle probe. The aggregate modulus, *H*_a_, was determined from the equilibrium stress–strain data as described by Mow and colleagues [5,6].

### Histological analysis

The cartilage samples were fixed in 10% formalin, decalcified in EDTA and then embedded in paraffin. Sagittal sections of 5 μm thickness were prepared from the center of the samples and stained with Safranin-O.

### *In vivo *study

The experimental OA model used in this study was created by intra-articular injection of collagenase into rabbit knee joints as reported by Kikuchi and colleagues [7]. Collagenase type ΙΙ (Worthington Biochemical Corporation) was dissolved in saline (530 U/ml), filtered with a 0.22 μm pore-size membrane, and used for the intra-articular injection. Japanese white adult rabbits (male, weight 3.0–5.5 kg; *n *= 24) were anesthetized with a mixture of ketamine (50 mg/ml) and xylazine (20 mg/ml) at a ratio of 2:1, by means of a dose of 1 ml/kg body weight injected intramuscularly into the gluteal muscle. After both knee joints had been shaved and sterilized, 0.5 ml of collagenase solution was injected intra-articularly into the right knee joint and/or saline was injected into the left knee joint as a control. The injection was performed twice, on days 1 and 4 of the experiment. The rabbits were returned to their cages and allowed to move freely without joint immobilization. For each experiment, four rabbits were killed at 0, 1, 4, 8, 12 and 16 weeks after the start of the experiment with an overdose of phenobarbital sodium salt, although two rabbits were discounted from the study because of a bacterial infection and a patellar dislocation, respectively. All the remaining knee joints were opened and the cartilage surfaces were observed macroscopically and photographed. The knee joint was dissected free from all soft tissues and the tibia was removed. The distal femur was cut proximally to the patellofemoral joint and cartilage samples were taken. Ultrasonic and biomechanical analyses were performed on the medial femoral condyle. For histological analysis, the lateral femoral condyles of the cartilage samples were fixed in 10% formalin, decalcified in EDTA and then embedded in paraffin. Sagittal sections of 5 μm thickness were prepared from the center of the samples and stained with Safranin-O. This study was approved by the Nara Medical University Ethics Committee.

### Statistical analysis

All data in this study are reported as means ± SD. The changes in the maximum magnitude, echo duration, water content, chondroitin sulfate content, hydroxyproline content and aggregate modulus with respect to the collagenase treatment duration were analyzed by one-way analysis of variance. Pearson correlations were performed to determine the associations between the ultrasonic data and the biochemical or biomechanical data. The significance level was set at *P *< 0.05.

## Results

### *In vitro *study

#### Ultrasonic measurement

**Figure 2:**
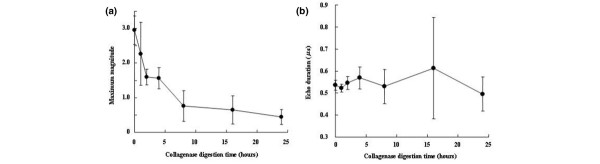
Time courses of the maximum magnitude (*P *< 0.01) **(a) **and echo duration (*P *= 0.14) **(b) **in collagenase-digested pig articular cartilage. Values are means ± SD.

The maximum magnitude decreased as the duration of collagenase digestion increased. There was a rapid decrease in the maximum magnitude after 8 hours of digestion in comparison with the control, and then a gradual decrease from 8 to 24 hours (Fig. 2a). There was no significant change in echo duration over the time course of digestion (Fig. 2b).

#### Biochemical measurement

**Figure 3:**
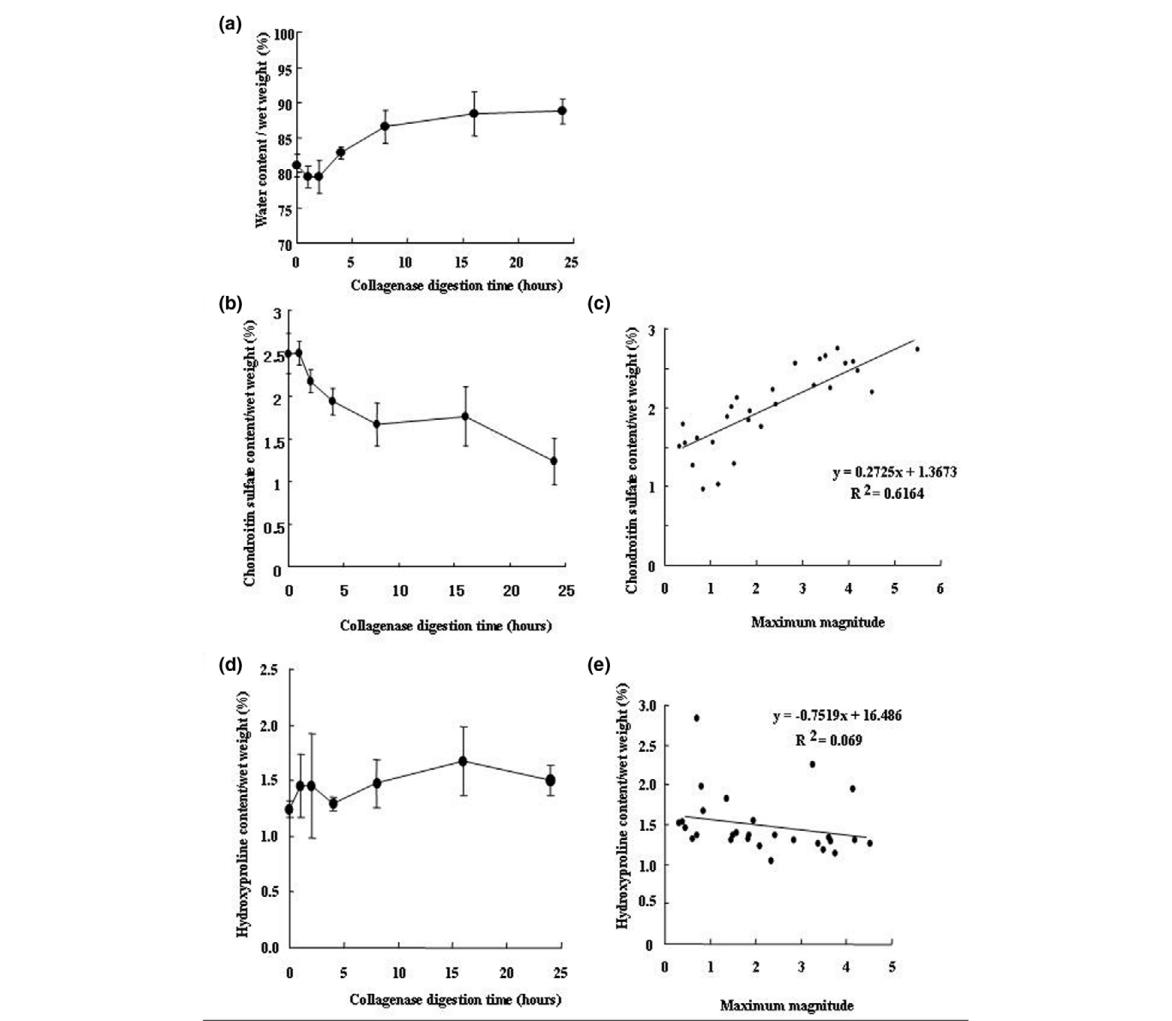
Time courses of the water content (*P *< 0.01) **(a)**, chondroitin sulfate content (*P *< 0.01) **(b) **and hydroxyproline content (*P *= 0.23) **(d) **in collagenase-digested articular cartilage. Values are means ± SD. The relationships between the maximum magnitude and the chondroitin sulfate content **(c) **and the maximum magnitude and the hydroxyproline content **(e) **are also shown.

The water content gradually increased over the time course of collagenase digestion (Fig. 3a). At the same time, the chondroitin sulfate content decreased rapidly with increasing duration of digestion. There was a rapid decrease in the chondroitin sulfate content after 8 hours of digestion and then a gradual decrease from 8 to 24 hours (Fig. 3b). There was a significant correlation between maximum magnitude and chondroitin sulfate content (*R*^2 ^= 0.6164, *P *< 0.01) (Fig. 3c). There was very little change in hydroxyproline content during collagenase digestion (Fig. 3d). There was no significant correlation between maximum magnitude and hydroxyproline content (*R*^2 ^= 0.069, *P *= 0.176) (Fig. 3e).

#### Biomechanical measurement

**Figure 4:**
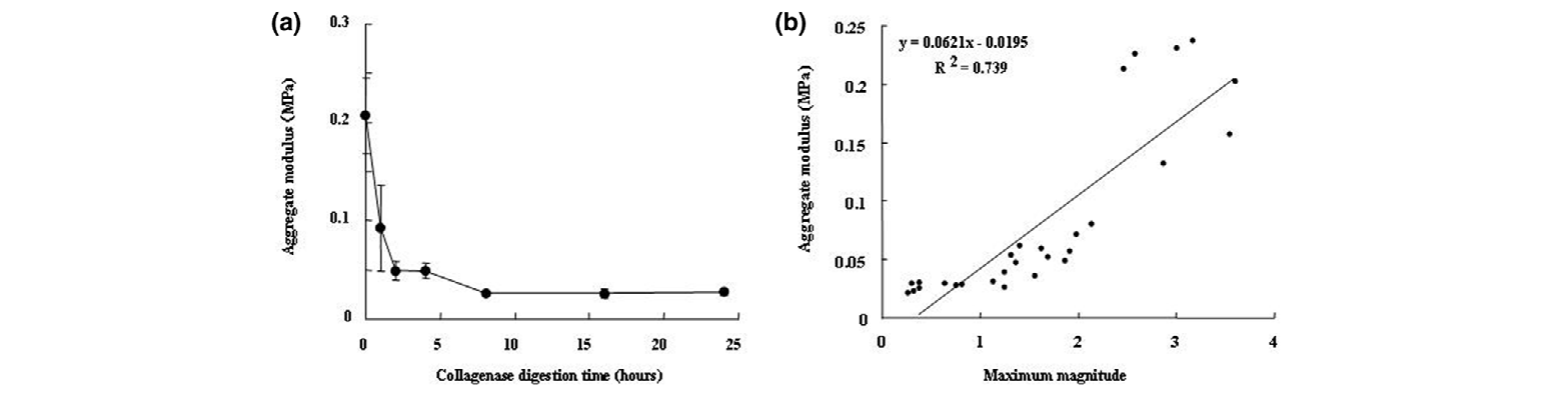
Time course of the aggregate modulus (*P *< 0.01) **(a) **in collagenase-digested articular cartilage. The relationship between the maximum magnitude and the aggregate modulus **(b) **is also shown.

The aggregate modulus rapidly decreased during the first 4 hours of collagenase digestion, but there was no subsequent change from 4 to 24 hours (Fig. 4a). There was a significant correlation between maximum magnitude and aggregate modulus (*R*^2 ^= 0.739, *P *< 0.01) (Fig. 4b).

#### Histological findings

**Figure 5:**
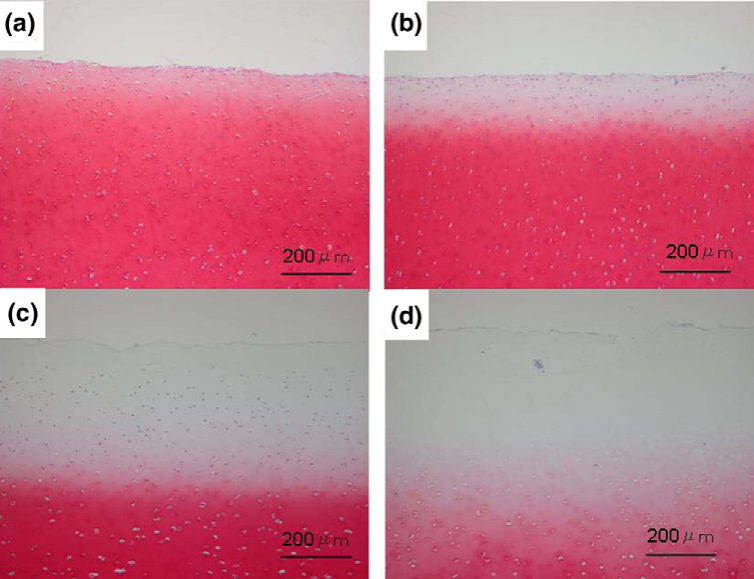
Photomicrographs of pig articular cartilage after 1 hour **(a)**, 4 hours **(b)**, 8 hours **(c) **and 16 hours **(d) **of collagenase digestion (Safranin-O stain; magnification × 4).

Representative sections of collagenase-digested cartilage stained with Safranin-O are shown in Fig. 5. In control cartilage, the Safranin-O staining of the extracellular matrix appeared almost homogeneous. After 1 hour of digestion, the superficial layer showed slight changes in the Safranin-O staining. After 8 hours of digestion, the surface layer to a depth of 500 μm was not stained with Safranin-O. Over the course of degeneration time, Safranin-O staining became less intense in the deeper layers.

### *In vivo *study

#### Macroscopic and histological findings

**Figure 6:**
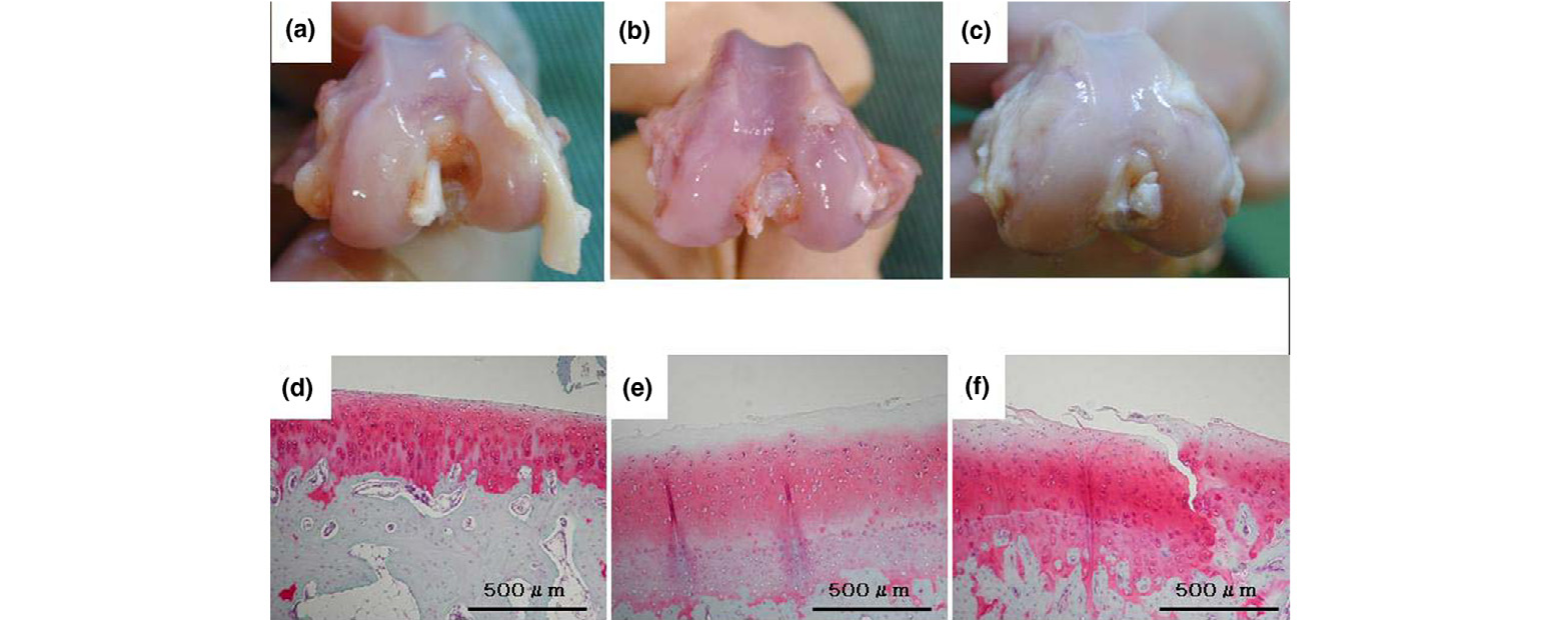
Macroscopic findings of rabbit articular cartilage at 1 week **(a)**, 4 weeks **(b) **and 8 weeks **(c) **after collagenase injection. Photomicrographs of rabbit articular cartilage at 1 week **(d)**, 4 weeks **(e) **and 8 weeks **(f) **after collagenase injection are also shown (Safranin-O staining; magnification × 4).

Figure 6 shows the macroscopic and histological findings of the collagenase-injected articular cartilage. Macroscopically, cartilage surface changes were not detected on either femoral condyle of the rabbits. Histologically, chondrocyte cluster formation was seen and the surface layer was not stained with Safranin-O at 4 weeks after injection. Several fissures were observed in the surface area at 8 weeks after injection.

#### Ultrasonic measurement

**Figure 7:**
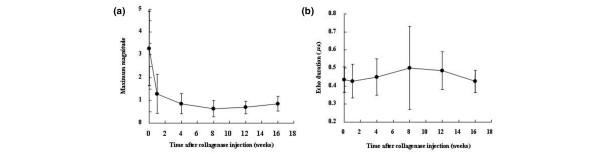
Time courses of the maximum magnitude (*P *< 0.01) **(a) **and echo duration (*P *= 0.55) **(b) **in collagenase-injected rabbit articular cartilage. Values are means ± SD.

The maximum magnitude decreased with increasing time after collagenase injection. There was a rapid decrease in the maximum magnitude at 4 weeks after injection, in comparison with control samples (Fig. 7a). However, there was no significant decrease in echo duration after injection (Fig. 7b).

#### Biomechanical measurement

**Figure 8:**
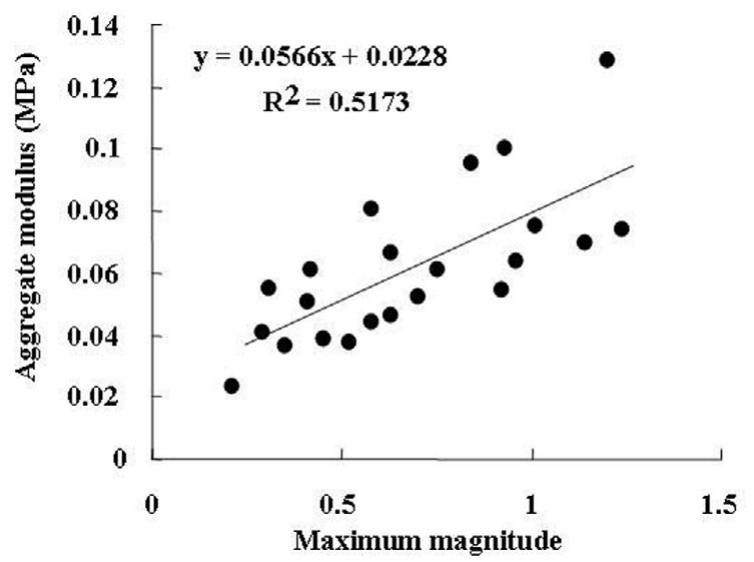
Correlation between the maximum magnitude and the aggregate modulus in collagenase-injected articular cartilage.

In the same manner as for the *in vitro *study, the relationship between the maximum magnitude and the aggregate modulus was investigated: there was a significant correlation (*R*^2 ^= 0.5173, *P *< 0.05) (Fig. 8).

## Discussion

The results of this study indicate that ultrasonic examination is promising as a minimally invasive method of evaluating microscopic damage in OA at an early stage. To evaluate microscopic damage to articular cartilage, reflex echoes from the cartilage were transformed into a wavelet map, and the echo duration and maximum magnitude were calculated and used as quantitative indices of cartilage degeneration. According to this study, the maximum magnitude was shown to reflect the proteoglycan content from biochemical analysis, the aggregate modulus from biomechanical analysis and the decrease in Safranin-O staining of the cartilage surface from histological analysis.

There are numerous clinical methods of grading the degenerative changes and injuries to articular cartilage at the time of surgery or arthroscopy with direct observation of the cartilage surface [8-10]. The overall observation from macroscopic findings and probing is that cartilage lesions vary in location, depth, size and shape. In addition, it is well established that probing cannot evaluate the cartilage condition quantitatively. As a quantitative method that could replace probing, attempts have been made to evaluate cartilage using magnetic resonance imaging, but such *in situ *evaluation has been performed only in experimental trials [11-13]. Cartilage biopsy and histological examination have been performed to evaluate articular cartilage clinically. However, it is still difficult to measure the degree of cartilage degeneration in a non-destructive manner. Therefore, further developments in diagnostic techniques are required for *in situ *evaluation.

Several different approaches have been investigated to improve the techniques for diagnosing the condition of cartilage, including optical coherence tomography [14], electromechanical evaluation [15], mechanical indentation [16], ultrasonic evaluation [17,18] and ultrasonic indentation [19-21]. Most of these approaches are still under development and only a few devices have been used successfully for cartilage evaluation during clinical investigations.

Ultrasonic indentation methods are capable of determining the cartilage thickness and deformation, and can therefore be used to determine the Young's modulus of articular cartilage. In a clinical context, Lyyra and colleagues [19] reported the efficacy of an ultrasonic indentation instrument under arthroscopic control for the quantification of cartilage stiffness, as evaluated with three human cadaver knees. This might prove to be suitable for clinical use, but the rod of the instrument (5 mm in diameter) is too thick to evaluate the cartilage in all regions of knee joints or the cartilage in ankle and wrist joints [21]. In contrast, our ultrasonic probe is so small (4 mm wide and 2.5 mm thick) that we can evaluate living human joint cartilage under arthroscopy. Moreover, we have reported clinically relevant data obtained from living human cartilage *in situ *[4].

Ultrasonic measurement under arthroscopy has three merits in comparison with arthroscopic indentation. The first is that the possibility of tissue damage caused by the measurement device can be completely excluded owing to the non-contact measurement. The second is that the evaluation system can predict the histological findings of cartilage on the basis of studies in experimental animal models [22,23]: hyaline cartilage has a higher maximum magnitude than fibrous tissue, whereas imperfectly regenerated cartilage has a lower maximum magnitude, even when only fibrous tissue and fibrocartilage are present in the superficial layer of the repaired tissue. The third is that the ultrasonic probe used in the evaluation is so small that it should be useful not only for articular cartilage in the knee joint but also for that in the wrist and ankle joints under arthroscopy.

Before this investigation, the maximum magnitude and echo duration were used as quantitative indices of degenerated cartilage, but it was not known what the indices were closely related to [3]. However, this study using a collagenase-induced OA model clarified the significance of the maximum magnitude. From an acoustic point of view, the maximum magnitude is a modification of the echo reflection from the cartilage surface, and hence differences in the surface reflection indicate significant alterations in the acoustic impedance among degenerated cartilage samples. From the histological findings, the matrix staining of the surface layer to a depth of 500 μm was closely related to the maximum magnitude. From a biochemical point of view, the proteoglycan content was more related to the maximum magnitude than the type ΙΙ collagen content. The collagen content showed little change after collagenase digestion in this study, although the collagen meshwork is widely known to be the main reflector of ultrasound and the source of ultrasound backscatter [24-26]. However, the apparent inconsistency between these observations and our results would be due to differences between the reflex echoes from flat ultrasound and focal ultrasound. From a biomechanical point of view, the maximum magnitude was related to the aggregate modulus from the mechanical properties of the articular cartilage. Therefore, the maximum magnitude reveals microstructural changes in degenerated cartilage and can provide diagnostically important information about the degenerated cartilage.

In this study, the echo duration showed no change over the time course of collagenase digestion. From the histological findings, the cartilage surface was smooth after collagenase digestion in the *in vitro *study and had several fissures only at 8 weeks after the collagenase injection. According to the previous human cadaver study, the echo duration becomes longer with macroscopic roughening of the cartilage surface due to wear [3]. Moreover, Myers and colleagues showed that the width of the echo band can be related to the depth of fibrillation in the macroscopic degenerative cartilage surface [27]. The echo duration is therefore closely related to the macroscopic fibrillation of articular cartilage.

There are three limitations to this study. First, the cartilage samples in this study were not human OA cartilage but collagenase-treated articular cartilage. However, OA-like changes were observed in the experimental animals after induction by intra-articular injection of collagenase, and enzyme-induced OA models are also used to investigate the pathogenesis of OA. Second, our evaluation system could not detect any microscopic roughness of the articular cartilage by using the index of echo duration. To detect this histological change, high-frequency ultrasound might be required. Finally, we did not detect the progression of cartilage degeneration in living humans. However, we have reported relevant clinical acoustic data from human cartilage *in situ *under arthroscopy. Further studies are therefore needed to determine whether this evaluation system will be beneficial for studying the pathogenesis of OA.

## Conclusion

Ultrasonic evaluation using a wavelet map can support the evaluation of microscopic damage of articular cartilage in OA. The evaluation system is suitable for clinical use under arthroscopy. This evaluation successfully predicted the histological findings of degenerated cartilage with the use of a collagen-induced OA model. We believe that our findings offer the potential for standardized evaluation as an adjunct to further research in this field, which will lead to a reliable method for the quantification of articular cartilage treatments.

## Abbreviations

OA = osteoarthritis.

## Competing interests

The author(s) declare that they have no competing interests.

## Authors' contributions

KH conceived the study, participated in its design and performed all the experiments. KI and YM performed biomechanical studies. YT participated in the design of the study and participated in the *in vivo *study. All authors read and approved the final manuscript.
